# Supplementary material for: The Global Impact of Hepatitis B Vaccination on Hepatocellular Carcinoma
Source: Vaccines (Basel). 2022 May 17;10(5):793. doi: 10.3390/vaccines10050793 (PMC9144632; doi:10.3390/vaccines10050793)
Supplement: Supplementary file 1 [file vaccines-10-00793-s001.zip › vaccines-1701008-supplementary.pdf]

Supplementary Table S1. Summary of studies assessing the impact of the hepatitis B vaccination schedule on HCC related outcomes.

Author	Year	Country/ Region	Study Type *	Intervention Years	Vaccinated Group: Age Range (Birth Year Cohort)	Unvaccinated Comparison Group/s, Age Range (Birth Year Cohort)	HCC Related Outcomes
Chang et al [15]	1997	Taiwan	O	1984 HBsAg mothers<br>1986 universal	N/A	N/A	Liver cancer cases in children aged 6–14 years from National Cancer Registry and Multi-centre childhood HCC and National mortality registry registration study 1981–1994:<br>• Average incidence (/100,000) of HCC declined (trend $p < 0.01$)<br>o 1981–1986: 0.7 (range 0.65–0.78)<br>o 1986–1990: 0.57 (range 0.48–0.62)<br>o 1990–1994: 0.36 (range 0.23–0.48)<br>• Incidence rate ratio 1986–1990/1981–1986 0.63<br>• Age adjusted RR of HCC after July 1990 compared to before was 0.33 ($p < 0.001$)<br>• Age-adjusted risk of death after July 1990 compared to before 0.51 ($p < 0.001$)
Lee et al [16]	2003	Taiwan	O	1984 HBsAg mothers<br>1986 universal	0–14 years	15–100 years	HCC mortality data between 1974–1999:<br>• Increase in mortality trend from 1974–1983 in age group 0–14<br>o Males: Spearman's coefficient 1, $p < 0.05$<br>o Females: Spearman's coefficient 0.5, $p > 0.05$<br>• Decrease in mortality trend from 1983 to 1999 in age group 0–14<br>o Males: Spearman's coefficient −1, $p < 0.05$<br>• Females: Spearman's coefficient −0.9, $p < 0.05$<br>Increase in mortality trend from 1983 to 1999 in reference age group 15–100 y<br>o Males: Spearman's coefficient 1, $p < 0.05$<br>o Females: Spearman's coefficient 0.5, $p > 0.05$<br>• In age group 0–14 years:

							○ Male mortality decreased by 70% in 1996–1999 compared to 1980–1983<br>○ Female mortality decreased by 62%% in 1996–1999 compared to 1980–1983
Chang et al [17]	2009	Taiwan	O	1984 HBsAg mothers 1986 universal	6–19 (1985–2004)	6–19 (1953–1984)	HCC incidence from July 1983 to June 2004:<br>• Overall RR 0.31 of HCC development in vaccinated group compared to unvaccinated group<br>• Incidence rates of HCC in unvaccinated age groups (/100,000):<br>○ 6–9 years: 0.49<br>○ 10–14 years: 0.56<br>○ 15–19 years: 0.60<br>○ 20–24 years: 1.07<br>○ 25–29 years: 2.28<br>• Incidence rates of HCC in vaccinated age groups (/100,000):<br>○ 6–9 years: 0.15<br>○ 10–14 years: 0.19<br>○ 15–19 years: 0.16<br>• Rate ratios of HCC development in vaccinated age groups compared to unvaccinated age groups (all $p < 0.001$)<br>○ 6–9 years: 0.3 (95% CI 0.18–0.42)<br>○ 10–14 years: 0.32 (95%CI 0.21–0.49<br>○ 15–19 years: 0.30 (95% CI 0.16–0.58)<br>• Incomplete vaccination associated with OR 4.32 for HCC development compared to complete vaccination (95%CI 2.34–7.91, $p < 0.001$)
Chang et al [18]	2016	Taiwan	O	1984 HBsAg mothers 1986 universal	6–26 (1984–2005)	6–26 (1956–1984)	HCC incidence rates in population aged 6–26 years diagnosed between June 1983 and June 2011<br>• Overall RR 0.24 of HCC development in vaccinated group compared to unvaccinated group<br>• Overall incidence rate of 0.92/100,000 person years in unvaccinated group compared to 0.23/100,000 in vaccinated group<br>• Incidence rate ratios for vaccinated/unvaccinated age groups (all $p < 0.0001$)

							○ 6–9 years: 0.26 (95% CI 0.17–0.40)<br>○ 10–14 years: 0.34 (95%CI 0.25–0.48)<br>○ 15–19 years: 0.36 (95% CI 0.25–0.51)<br>○ 20–26 years: 0.42 (95%CI 0.32–0.56)
Hung et al [19]	2015	Taiwan	O	1984 HBsAg mothers 1986 universal	82,856 people diagnosed with HCC	N/A	HCC prevention effect of different birth cohorts:<br>• Age standardised rate of 32.97/100,000 person years<br>• Proportion of HCC diagnosed per age group<br>○ Children (0–14 years): 0.04%<br>○ Adolescents and young adults (15–29 years): 0.8%<br>○ Middle aged (30–64 years): 50.1%<br>○ Elderly ≥ 65 years): 49.1%<br>• Annual percentage change in age standardised incidence rates for age groups from 2003 to 2011 (all $p < 0.05$):<br>○ Children: −16.6% (95% CI −29.7, −1.0,)<br>○ Adolescents and young adults: −7.9% (95%CI −10.0, −5.7)<br>○ Middle aged: −2.0% (95%CI −2.8, −1.1)<br>○ Elderly: 1.3% (95%CI 0.6, 1.9)
Chien et al [20]	2014	Taiwan	O	1984 HBsAg mothers 1986 universal	3,836,988 vaccinees from July 1984–March 2000	N/A	HCC incidence<br>• Incidence rate of HCC development (per 100,000 years) according to maternal HBsAg/HBeAg status<br>○ HBsAg (−)/HBeAg(−): 0.027<br>○ HBsAg (+)/HBeAg(−): 0.162<br>○ HBsAg (+)/HBeAg(+): 0.786<br>• Adjusted HR (95% CI) according to maternal HBsAg/HBeAg status<br>○ HBsAg (−)/HBeAg(−): 1.00 (ref)<br>○ HBsAg (+)/HBeAg(−): 3.9 (1.68–9.04, $p = 0.0015$)<br>○ HBsAg (+)/HBeAg(+): 17.58 (9.41–32.84, $p < 0.0001$)<br>• Incidence rate of HCC development (per 100,000 years) in mothers HBsAg (−)/HBeAg(−)<br>○ Vaccination complete: 0.099<br>○ Vaccination incomplete: 0.444

							• Incidence rate of HCC development (per 100,000 years) in mothers HBsAg (+)/HBeAg(+)<br>○ Ig administered: 0.578<br>○ Ig not administered: 1.39<br>• Gender adjusted HR (95% CI) compared to complete vaccination in HBsAg (−)/HBeAg(−) mothers<br>○ Vaccination incomplete: 4.4 (1.42–13.65) $p = 0.0103$<br>○ Ig administered: 5.51 (2.51–12.080 $p < 0.0001$)<br>○ Ig not administered: 12.71 (5.6–28.81) $p < 0.0001$)
Liao et al [21]	2021	Taiwan	O	1984 HBsAg mothers 1986 universal	1984–1994 period 2 1995–2003 Period 3 2004–2016 period 4	1979–1983 period 1	HCC incidence<br>• Overall HCC incidence increased<br>• HCC incidence RR in individuals aged <30 across the periods compared to period 1 since universal vaccination<br>○ Period 2 (post universal vaccination): 1.15 (95% CI 1.00–1.33)<br>○ Period 3 (post universal healthcare and screening ultrasounds): 1.4 (95% CI 1.2–1.62)<br>○ Period 4 (post national viral hepatitis treatment and surveillance program introduced): 0.9 (95% CI 0.76–1.05)<br>• HCC incidence RR in period 2 compared to period 1 in individuals aged 10–29 years: 1.49 (95% CI 1.31–1.7, $p < 0.0001$)
Wang et al [22]	2020	Guangxi, China	O	1986 LongAn county 2002 BinYang county	LongAn county 2017–2018	BinYang county 2017–2018 Non-vaccinated at birth in LongAn county 2004	HCC related mortality in 2017–2018:<br>• HCC mortality used as surrogate for HCC incidence<br>• Age adjusted mortality rate (per 100,000) ($X^2 = 7.9462$, $p = 0.005$)<br>○ LongAn: 53.3<br>○ BinYang 45.3<br>• No HCC case in either county in age group 0–19 years<br>• Mortality rate (per 100,000) in males ages 20–29 ($X^2 = 0.174$, $p = 0.667$)<br>○ LongAn: 2.7 (range 2.5–2.8)<br>○ BinYang: 4.7 (range 4.6–4.8)<br>○ RR 1.7<br>• Mortality rate (per 100,000) in males ages ≥30 years ($X^2 = 1.609$, $p = 0.032$)

							○ LongAn: 133.5 range 132.2–134.8)<br>○ BinYang: 116 (range 115.4–117)<br>○ RR 0.9<br>• Mortality rate (per 100,000) in LongAn in age group 20–29 ($X^2 = 5.554$, p = 0.018)<br>○ 2004: 7.9 (range 4.4–11.4)<br>○ 2017–2018: 1.4 (range 0.4–2.4)<br>• Mortality rate (per 100,000) in LongAn in age group ≥30 ($X^2 = 0.0412$, p = 0.839)<br>○ 2004: 97 (range 90.6–104.5)<br>○ 2017–2018: 95.9 (91.3–100.4)
Qu et al [23]	2014	Qidong, China	C	1985	Vaccinated towns<br>N = 38,366	Unvaccinated towns<br>N = 34,441	HCC incidence in children born in 1985–1990 in 41 rural towns across six clusters<br>• Primary liver cancer incidence rates (per 100,000)<br>○ Vaccinated towns: 0.21<br>○ Unvaccinated towns: 1.41<br>HR in vaccinated towns: 0.16 (p = 0.0224)<br>• Protective efficacy of vaccination 84%
The Gambia Hepatitis Study Group [24]	1987	The Gambia, Africa	P, O	1979<br>EPI<br>1986–1990<br>Sequential randomisation<br>1990<br>Universal vaccination	Vaccinated 1986–1990	Unvaccinated 1986–1990	For revaluation of protective effect of vaccination on HCC and CLD of children born during period of 1986–1990 when stepped wedge design of sequential randomisation of EPI teams every three months over four year period until all EPI teams administering HBV vaccine with other vaccinations.<br>Long term follow up through the national cancer registry continues.<br>Outcomes not available at time of publication.
Viviani et al [25]	2008	The Gambia, Africa	P, O	1979–1979<br>EPI<br>1986–1990<br>Sequential randomisation	N/A	N/A	65% subjects available for follow up<br>With expected cumulative incidence based on age specific HCC incidence rates from 1987–2002, final outcome for detecting significant impact of vaccination on HCC development will be measurable between 2017–2020 when subjects are approximately 30 years old.<br>Outcomes not available at time of publication

McMahon, et al [26]	2011	Alaska, United States of America	R, O	1990 Universal vaccination<br><br>1984 Alaska Native Hepatitis B Control Program	Children <20 years	N/A	HCC incidence identified by national cancer institute cancer registry and HCC surveillance program set up by Liver Disease and Hepatitis Program between 1969 and 2008:<br>• Incidence of HBV-HCC in 1970s was high with 1/3 HCC cases occurring in <30 years<br>• Annual incidence of HCC (/100,000) in children aged <20 years ($p < 0.001$ for overall trend)<br>○ 1984–1988 (peak): 3<br>○ 1999: 0

* O, observational; R, retrospective; P, prospective; C, cluster randomised controlled trial.
